# Complete Mitogenomes of Ticks *Ixodes acutitarsus* and *Ixodes ovatus* Parasitizing Giant Panda: Deep Insights into the Comparative Mitogenomic and Phylogenetic Relationship of Ixodidae Species

**DOI:** 10.3390/genes13112049

**Published:** 2022-11-06

**Authors:** Jiabin Liu, Jiaojiao Yu, Xiang Yu, Wenlei Bi, Hong Yang, Fei Xue, Gexiang Zhang, Jindong Zhang, Dejiao Yi, Rui Ma, Yanshan Zhou, Guanwei Lan, Jiang Gu, Wei Wu, Zusheng Li, Guilan Qi

**Affiliations:** 1 Sichuan Key Laboratory of Conservation Biology for Endangered Wildlife, Chengdu Research Base of Giant Panda Breeding, Chengdu 610081, China; 2 Management Center of Daxiangling Nature Reserve in Yingjing County, Ya’an 625200, China; 3 College of Computer Science and Cyber Security, Chengdu University of Technology, Chengdu 610059, China; 4 Key Laboratory of Southwest China Wildlife Resources Conservation (Ministry of Education), China West Normal University, Nanchong 637009, China; 5 Chengdu Academy of Agriculture and Forestry Sciences, Chengdu 611130, China

**Keywords:** giant panda, mitogenome, Ixodidae, comparative mitogenomics, mitochondrial phylogenomics

## Abstract

Ticks rank second in the world as vectors of disease. Tick infestation is one of the factors threatening the health and survival of giant pandas. Here, we describe the mitogenomes of *Ixodes acutitarsus* and *Ixodes ovatus* parasitizing giant pandas, and perform comparative and phylogenetic genomic analyses on the newly sequenced and other available mitogenomes of hard ticks. All six newly determined mitogenomes contain a typical gene component and share an ancient Arthropoda gene arrangement pattern. Our study suggests that *I. ovatus* is a species complex with high genetic divergence, indicating that different clades of *I. ovatus* represent distinct species. Comparative mitogenomic analyses show that the average A + T content of Ixodidae mitogenomes is 78.08%, their GC-skews are strongly negative, while AT-skews fluctuate around 0. A large number of microsatellites are detected in Ixodidae mitogenomes, and the main microsatellite motifs are mononucleotide A and trinucleotide AAT. We summarize five gene arrangement types, and identify the *trnY*-*COX1*-*trnS1*-*COX2*-*trnK*-*ATP8*-*ATP6*-*COX3*-*trnG* fragment is the most conserved region, whereas the region near the control region is the rearrangement hotspot in Ixodidae mitogenomes. The phylogenetic trees based on 15 genes provide a very convincing relationship (*Ixodes* + (*Robertsicus* + ((*Bothriocroton* + *Haemaphysalis*) *+* (*Amblyomma* + (*Dermacentor* + (*Rhipicentor* + (*Hyalomma* + *Rhipicephalus*))))))) with very strong supports. Remarkably, *Archaeocroton sphenodonti* is embedded in the *Haemaphysalis* clade with strong supports, resulting in paraphyly of the *Haemaphysalis* genus, so in-depth morphological and molecular studies are essential to determine the taxonomic status of *A. sphenodonti* and its closely related species. Our results provide new insights into the molecular phylogeny and evolution of hard ticks, as well as basic data for population genetics assessment and efficient surveillance and control for the giant panda-infesting ticks.

## 1. Introduction

Ticks, which are common hematophagous ectoparasites, are capable of parasitizing almost all groups of terrestrial vertebrates, including mammals, birds, lizards, snakes, and toads [1,2,3]. Ticks rank second in the world as vectors of disease, and they can harbor and transmit a great variety of pathogens, including virus, bacteria, fungi, protozoa, and nematode, to humans, livestock, and wildlife [2,4,5,6]. More than 28 species of ticks are known to cause a variety of human diseases [5,7]. Tick-borne diseases cause severe illness and even death in animals and humans, resulting in significant loss of life and property [5,8,9]. Ticks and tick-borne diseases are a serious public health concern and have drawn heightened attention from clinicians and researchers.

The giant panda (*Ailuropoda melanoleuca*) is one of the most recognized flagship and umbrella species for biodiversity conservation in the world, and its conservation work is a showcase program for the Chinese government and its collaborators and has attracted worldwide attention [10,11,12]. Currently, there is a large population of giant pandas, including 1864 wild individuals living in 2.58 million hectares of habitat and 673 captive individuals. However, the development of giant panda population is still hampered by many factors such as infectious diseases [13,14,15,16]. There is still a lot of work to do to further consolidate the achievements of giant panda conservation.

The diseases caused by parasites have major impacts on the health and survival of giant pandas [15]. Up to now, 35 parasites species have been identified from giant pandas, and 13 of them are hard ticks (Ixodidae, including nine *Haemaphysalis* species, three *Ixodes* species, and one *Dermacentor* species) [17]. The infection rate of ticks in giant pandas is very high, even up to 100%, both in the wild and in captivity [17]. Ticks can cause even more damage by spreading pathogens, such as protozoans and viruses. The *Babesia* sp., one of the most widespread tick-borne protozoan parasites that can cause Babesiosis, is detected in the blood of giant panda [18]. Thirty-two viral species are detected in the giant panda-infesting ticks, half of which show homology to viruses carried by giant pandas and their associated host species [19]. More seriously, the mixed parasitism of a variety of ticks is common on giant pandas [17,20].

Correct identification of tick species is essential for the study of ticks and tick-borne pathogens [21]. The conventional method for identifying ticks is based on microscopic observation of morphological features, which is suitable for adult ticks [3]. Many tick species share great morphological similarities and therefore group into species complexes until differentiating characters can be described [22]. The mitochondrial genome (mitogenome) is a reliable marker for cryptic species identification, phylogenetic studies, and population genetics analysis, and has been widely used in animals [23,24,25,26,27,28], including in ticks [7,21,29]. In recent years, the number of sequenced mitogenomes of Ixodidae species has increased significantly, but the mitogenome studies on parasitic ticks of giant pandas have only been performed for three *Haemaphysalis* species [30,31,32]. The mitogenomes of another ten Ixodidae species infesting giant pandas have not been reported. Our knowledge of the genetic diversity of these ticks is lacking. Recently, we found that our wild-training giant panda individuals living in Yingjing County of Sichuan Province were also infected with ticks, which were identified as *I. acutitarsus* (Karsch, 1880) and *I. ovatus* Neumann, 1899 by morphological and preliminary molecular identification. Cases of giant pandas infected with these two ticks have been reported in Tianquan, Baoxing, and Wenchuan of Sichuan, and Wenxian of Gansu [17].

Comparative mitogenomic analyses are of great significance for understanding the biodiversity and evolution, and mitogenomic data can provide efficient phylogenetic signals for a wide variety of animal groups [3,24,33,34]. The phylogenetic relationships of hard ticks have been studied in depth [3,4,35,36,37,38,39], but comprehensive comparative mitogenome analyses of them are rarely performed. Herein, we sequenced the mitogenomes of the giant panda-infecting ticks *I. acutitarsus* and *I. ovatus* through high-throughput sequencing technology, and performed comparative and phylogenetic genomic analyses for the newly sequenced and publicly available mitogenomes of hard ticks.

## 2. Materials and Methods

### 2.1. Specimen Collection and DNA Extraction

Our team is developing a program to release captive-bred giant pandas into the wild by adopting the Human-Assisted Soft Release Method. Through this method, our investigators can examine the trained giant pandas and collect more data in the wild without undergoing anesthesia [19,40]. A total of eight adult ticks were collected from the body surface of our wild-training giant pandas living in Yingjing Area of Giant Panda National Park (102°19′30″ E–102°57′0″ E, 29°28′30″ N–29°57′0″ N) from March to April 2022, and stored in 95% ethanol. These ticks were identified as *I. acutitarsus* (specimen numbers PB2022006–PB2022009) and *I. ovatus* (specimen numbers PB2022017–PB2022020) according to morphological characters and nucleotide BLAST results of *ITS2* and *COX1* genes. Two of them (specimen numbers PB2022009 and PB2022020) were kept as voucher specimens and deposited at Chengdu Research Base of Giant Panda Breeding, and the genomic DNA of the remaining six ticks were extracted using the DNeasy Blood and Tissue Kit (QIAGEN, Hilden, Germany).

### 2.2. Library Preparation and Mitogenome Sequencing

Genomic DNA samples were sent to Sangon Biotech Co., Ltd. (Shanghai, China) for High-throughput sequencing. For each sample, approximately 300 ng genomic DNA were sheared into fragments of 400–500 bp using Covaris S220 system (Covaris, Woburn, MA, USA), and used for Illumina sequencing libraries preparation using Hieff NGS MaxUp II DNA Library Prep Kit for Illumina (YEASEN, Shanghai, China). Sequencing was performed on Illumina Novaseq 6000 platform (Illumina, San Diego, CA, USA) with a paired-end read length of 150 bp according to the standard protocols, and sequencing reads (raw data) were exported to the FASTQ format.

### 2.3. Mitogenome Assembly and Annotation

The raw data were processed for quality control and preprocessing using fastp v0.36 [41]. The high-quality clean data were assembled into contiguous sequences using SPAdes v3.15 with different *K*-mer parameters [42]. On the basis of NCBI BLAST searches, the contigs mapped to tick mitochondrial genome sequence were used for generating the circular mitogenomes of the two examined *Ixodes* species.

Mitogenome annotation was performed as previously described [33]. MITOS WebServer (http://mitos2.bioinf.uni-leipzig.de/index.py, accessed on 25 July 2022) [43] was used to predetermine protein-coding genes (PCGs), ribosomal RNA genes (rRNAs), and transfer RNA genes (tRNAs) and define their respective gene boundaries based on invertebrate mitochondrial genetic code. PCGs were reconfirmed using NCBI ORFfinder (http://www.ncbi.nlm.nih.gov/gorf/gorf.html, accessed on 25 July 2022) [44], and tRNAs were rechecked using tRNAscan-SE web server (http://lowelab.ucsc.edu/tRNAscan-SE/, accessed on 25 July 2022) [45] and ARWEN online services (http://130.235.244.92/ARWEN/, accessed on 25 July 2022) [46] based on their proposed cloverleaf secondary structures and anticodon sequences. As a further step toward improving the mitogenome annotations, all fragments were aligned with other *Ixodes* species’ homologs (e.g., *I. granulatus* OM368258, OM368272; *I. ovatus* OM317739). Finally, OGDRAW v1.3.1 (https://chlorobox.mpimp-golm.mpg.de/OGDraw.html, accessed on 25 July 2022) [47] was used to visualize the mitogenome organization.

### 2.4. Available Mitogenome Retrieval

We searched Ixodidae sequences on GenBank (https://www.ncbi.nlm.nih.gov/genbank/, accessed on 28 July 2022) based on keyword ‘Ixodidae’, and obtained 479 initial results by setting sequence length to ‘10,000–30,000 bp’ and selecting the ‘mitochondrion’ sequence. Among them, 128 results were filtered out due to incomplete sequencing or lacking annotation information, and 351 mitogenome sequences were finally selected for subsequent analysis (Table S1). We retrieved these 351 Ixodidae mitogenomes from GenBank using PhyloSuite v1.2.2 [48].

### 2.5. Comparative Mitogenomic Analyses

All 351 retrieved mitogenomes belonging to 107 species and 10 genera were used for comparative mitogenomic analyses (Table S1), in combination with our six newly determined mitogenomes. The sequences were aligned with MAFFT online service (https://mafft.cbrc.jp/alignment/server/, accessed on 28 July 2022) using ‘--auto’ strategy [49]. The nucleotide composition, variable sites statistics, and Kimura 2-parameters (K2P) genetic distance of the mitogenomes were calculated using MEGA v11 [50]. The following formulas were used to measure nucleotide composition bias: AT-skew = (A − T)/(A + T) and GC-skew = (G − C)/(G + C) [51]. Krait v1.3.3 [52] and MISA-web (https://webblast.ipk-gatersleben.de/misa/, accessed on 29 July 2022) [53] were used to screen the mitogenomes for microsatellite sequences. The minimum repeat numbers for each perfect microsatellite type were set to 10 for mono-, 5 for di-, 4 for tri-, 3 for tetra-, 3 for penta-, and 3 for hexa-nucleotides, respectively [54,55]. The microsatellite count per kilobase pair sequence was defined as the relative abundance (RA) of microsatellite and was measured by the following formula: RA = (total number of microsatellite loci)/(whole mitogenome size) × 1000 [52,54]. DnaSP v6.12.03 [56] was used to calculate the nucleotide diversity of rRNAs and PCGs, and conduct sliding window analysis of whole mitogenomes implementing window size of 200 bp and a step size of 20 bp. The qMGR web server (https://qmgr.hnnu.edu.cn/, accessed on 30 July 2022) [57] was used to conduct the gene arrangement analysis by calculating the rearrangement frequency (RF) of single gene and the total rearrangement score (RS) of every gene arrangement type in Ixodidae group, and the ancient Arthropoda gene arrangement type was chosen as the benchmark arrangement [24,58,59].

These data were visualized as graphs generated by ImageGP v1.0 (http://www.ehbio.com/Cloud_Platform/front/#/, accessed on 31 July 2022) [60] and OmicStudio tool (https://www.omicstudio.cn/tool, accessed on 31 July 2022).

### 2.6. Mitogenomic Phylogenetic Analyses

Many of the 351 retrieved Ixodidae mitogenomes belong to the same species (e.g., multiple representatives for *Amblyomma testudinarium*, *Haemaphysalis longicornis*, *Hyalomma marginatum*, and *I. ricinus*), so we only selected 226 representative mitogenomes belonging to 107 tick species and 10 genera to reconstruct phylogenetic relationships (Table S1), in combination with the six newly determined mitogenomes in this study. To root the trees, we used *Argas africolumbae* (Argasidae) and *Nuttalliella namaqua* (Nuttalliellidae) as outgroups according to the evolutionary relationships of the Ixodida [3,61,62].

Two rRNAs and 13 PCGs sequences from 234 mitogenomes were aligned in batches with MAFFT v7.313 [63] using ‘Normal’ or ‘Codon’ alignment mode and ‘--auto’ strategy, then trimmed gap sites with trimAl v1.2 [64] using ‘automated1′ command, and eventually concatenated into one multi-gene dataset consisting of 12,892 bp sequence, which was used for phylogenetic tree inference. The Bayesian Inference (BI) phylogenetic tree was constructed using MrBayes v3.2.6 [65] under a partition model (two parallel runs, ten million generations), in which the initial 25% of sampled data was discarded as burn-in. Maximum Likelihood (ML) phylogenetic tree was constructed using IQ-TREE v1.6.8 [66] under Edge-linked partition model for ten thousand replicates of ultrafast bootstraps [67], as well as the SH–aLRT branch test [68]. The best-fit Edge-linked partition substitution model was chosen by ModelFinder [69] using BIC criterion (Table S2). All of the above analyses were implemented in PhyloSuite v1.2.2 [48].

The phylogenetic trees were visualized by iTOL v6 (https://itol.embl.de/, accessed on 31 July 2022) [70].

## 3. Results and Discussion

### 3.1. Characterization of the Newly Sequenced Mitogenomes

#### 3.1.1. General Characteristics

**Figure 1:**
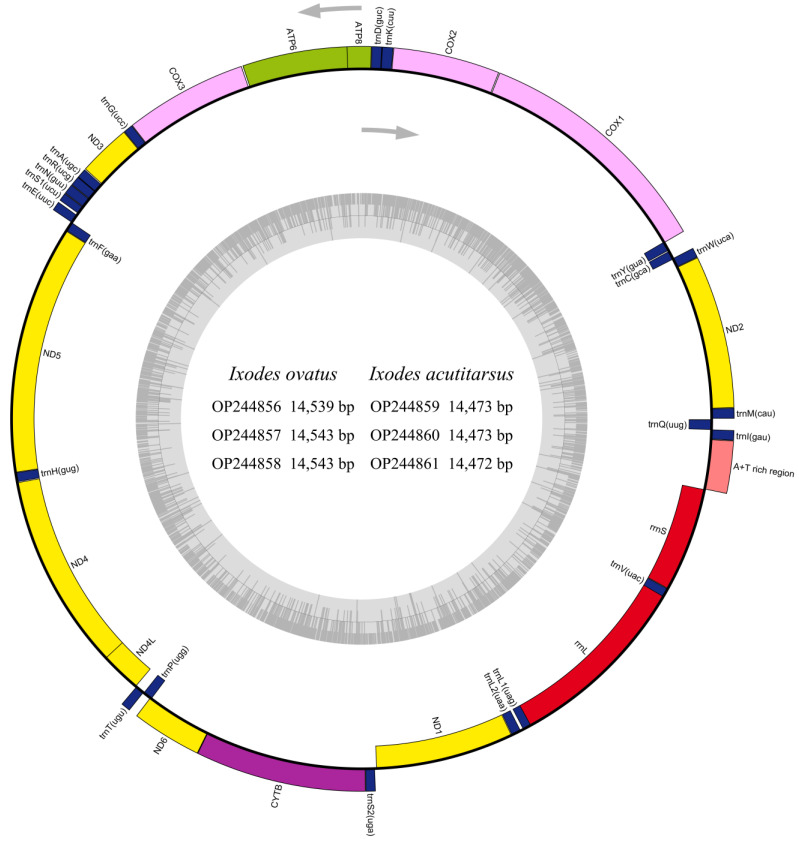
Structural representation of the mitogenomes of *I. ovatus* and *I. acutitarsus*. The arrows represent the transcription direction of genes on heavy (outside) and light (inside) chain of the mitogenomes. The tRNAs are abbreviated by the one-letter code for the corresponding amino acid, and the anticodons are indicated in parentheses. The inner circle is the G + C content graph of mitogenome OP244856.

We successfully sequenced and characterized six mitochondrial genomes of two hard tick species, *I. ovatus* and *I. acutitarsus*. The newly determined mitogenomes of *I. ovatus* were circular genomes with 14,539 bp, 14,543 bp, and 14,543 bp in size (Figure 1), which were larger than previously published mitogenomes of the same species (14,507–14,520 bp). The newly determined mitogenomes of *I. acutitarsus* were 14,472 bp, 14,473 bp, and 14,473 bp in size (Figure 1), which were the smallest complete mitogenomes of hard ticks so far.

These six mitogenomes shared the same gene content and arrangement, which was consistent with previous studies [59,62,71]. They had a typical gene content, including 22 tRNAs, 13 PCGs, two rRNAs, and one control region (CR, also named A + T rich region), of which 15 genes (*trnC*, *trnF*, *trnH*, *trnL1*, *trnL2*, *trnP*, *trnQ*, *trnV*, *trnY*, *ND1*, *ND4*, *ND4L*, *ND5*, *rrnL*, and *rrnS*) were transcribed from the light chain (Figure 1, Table S3). The gene arrangements of the six mitogenomes were the same as the ancient Arthropoda gene arrangement type [58]. In these mitogenomes, all 13 PCGs were started with the typical initiation codon ATA/ATT/ATC/ATG. *ATP6*, *ATP8*, *COX1*, *ND3*, *ND4*, *ND4L*, and *ND6* genes were terminated by the canonical termination codon TAA/TAG, while the other six genes (*COX2*, *COX3*, *CYTB*, *ND1*, *ND2*, and *ND5*) were ended with the truncated termination codon T (Table S3). The usage of incomplete stop codon T was a common feature in many metazoans [35,38,72,73,74], and it may be converted into complete termination codon TAA by polyadenylation after transcription [75].

The CR of *I. ovatus* and *I. acutitarsus* was located between *rrnS* and *trnI* (Figure 1). The *I. ovatus* CR was 333–334 bp in size, and its A + T content reached 71.77–71.86%, which was lower than the overall A + T content of the whole mitogenome (75.20–75.29%). The *I. acutitarsus* CR was only 343–344 bp in size, but it contained higher A + T content than the whole mitogenome (82.22–82.27% vs. 78.28–78.30%).

#### 3.1.2. Tick-Box Motif

Montagna et al. identified a 17 bp degenerate consensus sequence (5′-TTGyrTChwwwTwwGdA-3′) named Tick-Box motif in all tick mitogenomes, and found this motif was always present in only two or three fixed genomic positions: downstream of *ND1* (mainly in noncoding regions), near the 3′-end of *rrnL* (occasionally within *trnL1*), and/or inside small noncoding region located between *trnQ* and *trnF* in some Metastriata ticks [76]. In our newly determined and other publicly available mitogenomes of *I. ovatus* and *I. acutitarsus*, we found the identical motifs were located within *trnL1* (5′-TTGTATCAAATTTAGAA-3′) and downstream of *ND1* (5′-TTGTrTCCTTTTwAGAA-3′). Other research teams also detected the Tick-Box motifs in *Amblyomma americanum*, *Dendrobates auratus*, *D. silvarum*, *Ha. kitaokai*, *Ha. longicornis*, *Hy. marginatum*, *Hy. rufipes*, *Rhipicephalus microplus*, and many soft tick mitogenomes [37,38,62,77,78,79,80,81]. Although Lu et al. reported that no sequence identical to the Tick-Box consensus motif was present in *I. vespertilionis* mitogenome [82], we successfully found the motif within *trnL1* (5′-TTGTATCAAATTTAGAA-3′) and downstream of *ND1* (5′-TTGTATCCTTTTGAGAA-3′) on the light chain.

#### 3.1.3. Genetic Divergence, Nucleotide Variation, and Diversity

The K2P genetic distances were listed in Table 1 and Table 2. The genetic divergence between some individuals was very high. The recommended species boundaries for ticks were 0.0525 for *rrnL* and 0.0613 for *COX1* [83,84]. Based on this criterion, we considered *I. ovatus* and *I. acutitarsus* as two species complexes and divided seven *I. ovatus* individuals into four groups and five *I. acutitarsus* individuals into two groups. Recently, one study also found that *I. ovatus* was species complex with high genetic diversity [22], and a potential cryptic species was revealed in the *I. ovatus* group [7]. Therefore, all results suggested that different groups of *I. ovatus* might represent distinct species. Unfortunately, no between-group morphological comparisons were performed in our study. In-depth morphological comparisons and molecular studies need to be performed to resolve the taxonomic status of the *I. ovatus* and *I. acutitarsus* species complexes.

**Figure 2:**
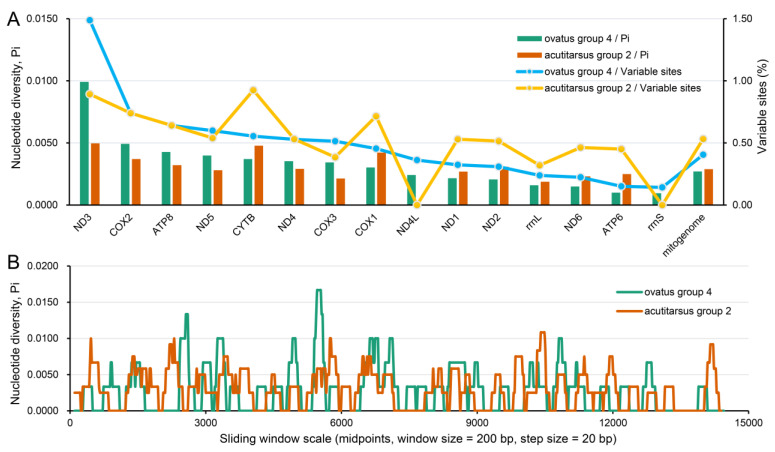
Nucleotide variation and diversity of *I. ovatus* group 4 and *I. acutitarsus* group 2 mitogenomes. (**A**) Variable nucleotide sites statistics and nucleotide diversity estimates of *I. ovatus* group 4 and *I. acutitarsus* group 2 mitochondrial genes. (**B**) Sliding window analysis of the complete mitogenome alignments between *I. ovatus* group 4 and *I. acutitarsus* group 2. *I. ovatus* group 4 include three mitogenomes with GenBank IDs OP244856–OP244858. *I. acutitarsus* group 2 include four mitogenomes with GenBank IDs OL800704, OP244859–OP244861.

Our six newly determined mitogenomes were grouped into the *I. ovatus* group 4 and *I. acutitarsus* group 2 (Table 1 and Table 2), respectively. For these two species groups, we calculated the nucleotide variable site percentage and the nucleotide diversity level. At the mitochondrial genome-wide level, the nucleotide variable site percentage and the nucleotide diversity level of *I. ovatus* group 4 were 0.41% and 0.0027, respectively, which were slightly lower than the corresponding values of *I. acutitarsus* group 2 (0.53% and 0.0029) (Figure 2A). Sliding window analysis of the mitogenomes showed a similar nucleotide diversity result between these two groups (Figure 2B). As shown in Figure 2A, at the mitochondrial single-gene level of these two species groups, *ND3* had the highest and *rrnS* had the lowest nucleotide variable site proportion, and the same situation also appeared at the nucleotide diversity level. Very low nucleotide diversity in *rrnS* was also found among nine *Ixodes* species [71].

### 3.2. Comparative Mitogenomic Analyses of Ixodidae Species

#### 3.2.1. Nucleotide Composition and Skewness

We calculated the nucleotide composition of 357 Ixodidae mitogenomes representing 107 species of 10 genera. The Ixodidae mitogenomes size averaged 14,752 bp, the longest reached 15,307 bp (*Dermacentor* sp. OM368308), and the smallest was only 14,472 bp (*I. acutitarsus* OP244861).

**Figure 3:**
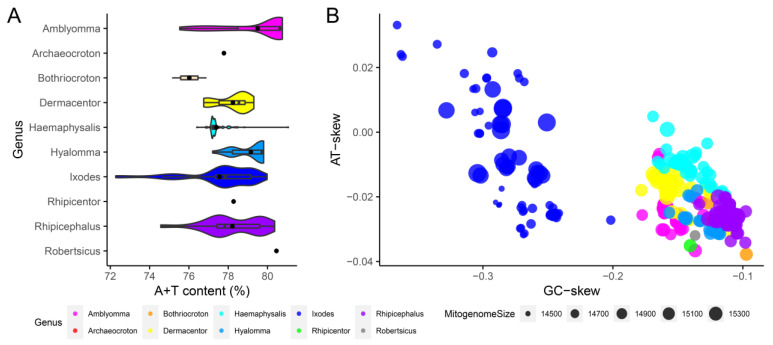
Nucleotide content and skewness of Ixodidae mitogenomes. (**A**) Intergenus comparison of A+T content of Ixodidae mitogenomes. (**B**) Intergenus comparison of AT-skew and GC skew of Ixodidae mitogenomes.

At the mitochondrial genome-wide level, most Ixodidae species had the characteristics of T% > A% > C% > G% except *Ha. montgomeryi* MW751681 and a few *Ixodes* species with the characteristics of A% > T% > C% > G% (Figure S1). In metazoan mitogenomes, A + T content is generally higher than G + C content [33,61,85,86,87,88]. Previous statistics indicated the A + T content of Ixodidae mitogenomes was over 70% [71]. Wang et al. also found the ticks (including Ixodidae, Argasidae and Nuttalliellidae) had high A + T content with a mean of 75.51%, and the A + T content in Ixodidae family was higher than in the Argasidae family [61]. In this study, we further accurately calculated that the average A + T content of Ixodidae mitogenomes was 78.08%, ranging from 72.28% (*Ixodes* sp. MW021452) to 81.06% (*Ha. danieli* OM368292). Such a high proportion of A + T content was also present in other arthropod groups, such as Araneae [34] and Lepidoptera [89]. Among 10 genera of the Ixodidae family, *Ixodes* genus had the largest A + T content variation range from 72.28% to 79.98%, and its average was 77.56%, which was smaller than 79.51% (75.53–80.76%) of the *Amblyomma* genus, 79.15% (77.20–79.80%) of the *Hyalomma* genus, 78.24% (76.75–79.30%) of the *Dermacentor* genus, 78.21% (74.57–80.37%) of the *Rhipicephalus* genus, and larger than 77.40% (76.40–81.06%) of the *Haemaphysalis* genus (Figure 3A).

Strand asymmetry of nucleotide composition is usually described as skewness and measured by AT-skew and GC-skew values [90,91]. As shown in Figure 3B, the AT-skews of Ixodidae mitogenomes fluctuated around 0 (−0.038–0.033) and averaged −0.017, revealing that the A content and T content were relatively close; the GC-skews were strongly negative (−0.366–−0.097), with the mean of −0.172, indicating a higher occurrence of C than G; the GC-skews of *Ixodes* species were smaller than those of non-*Ixodes* species. Generally, GC-skew of heavy chain of metazoan mitogenome was negative, while AT-skew was positive [74,91,92,93]. Apparently, the AT-skew of Ixodidae mitogenomes deviated from the general characteristics of metazoan mitogenomes. Negative AT-skews had also been found in the mitogenomes of other taxonomic groups, such as Lepidoptera [86,89] and Araneae [34]. Such skews towards a particular nucleotide can be related to asymmetric mutational constraints [92].

**Figure 4:**
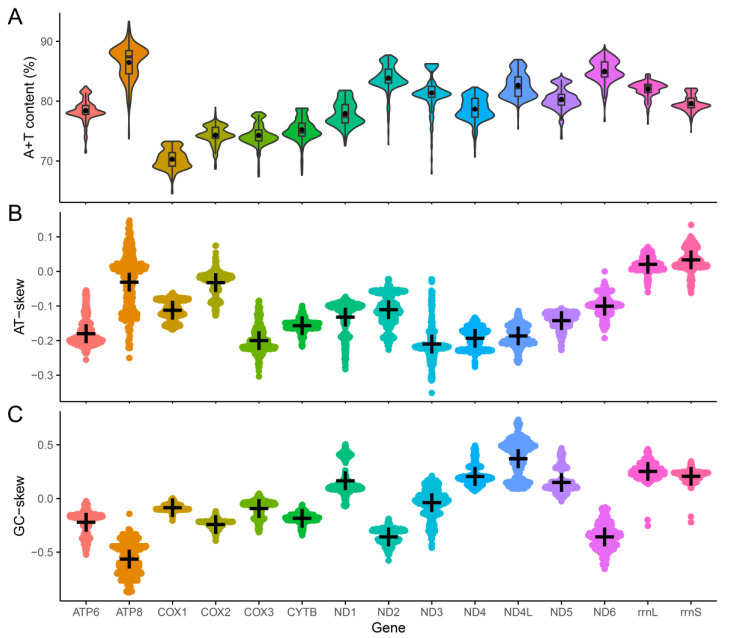
Nucleotide content and skewness of Ixodidae mitochondrial PCGs and rRNAs. (**A**) A+T content of Ixodidae mitochondrial genes. (**B**) AT-skew of Ixodidae mitochondrial genes. (**C**) GC-skew of Ixodidae mitochondrial genes.

We also counted the nucleotide composition of individual genes in these 357 Ixodidae mitogenomes. At the mitochondrial single-gene level (including 13 PCGs and two rRNAs), A + T content of Ixodidae species ranged from 70.31% (*COX1*) to 86.47% (*ATP8*), which was much larger than the G + C content (Figure 4A); AT-skews ranged from −0.209 (*ND3*) to 0.034 (*rrnS*) (Figure 4B); GC-skews ranged from −0.562 (*ATP8*) to 0.375 (*ND4L*) (Figure 4C); *ND1*, *ND4*, *ND4L*, *ND5*, *rrnL*, and *rrnS* genes transcribed by the light chain had higher GC-skew values than those genes transcribed by the heavy chain, while only *rrnL* and *rrnS* had higher AT-skews than other genes (Figure S2).

#### 3.2.2. Microsatellite Characteristics

**Figure 5:**
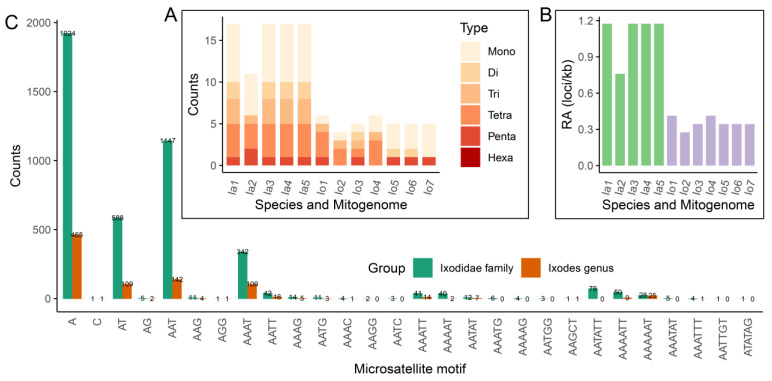
Statistics of microsatellite identified in Ixodidae mitogenomes. (**A**) The microsatellite loci counts and (**B**) their relative abundances of *I. acutitarsus* and *I. ovatus* species complex mitogenomes. Ia1–5 represent *I. acutitarsus* species complex mitogenomes with GenBank IDs OL800704, OM368264, and OP244859–OP244861; Io1–7 represent *I. ovatus* species complex mitogenomes with GenBank IDs OM317739, OM368266, OM368268, OM366269, and OP244856–OP244858. (**C**) The counts statistics of different microsatellite motif in Ixodidae and *Ixodes* mitogenomes.

Microsatellites, also known as Short Tandem Repeats (STRs) and Simple Sequence Repeats (SSRs), are tandem repeats of simple sequence motifs widely found in eukaryotic genomes [94]. They exhibit extensive polymorphism due to copy number variations of each specific repeat unit, and are considered an important type of repeated sequences that are popular as molecular markers for genetic diversity analysis [95]. Copy number variations of microsatellites in protein-coding genes can lead to frameshift mutations in proteins that can cause deleterious effects [96]. Microsatellites of nuclear genomes have been extensively studied, whereas surveys of microsatellites in organelle genomes are rare [54,55,86,96]. In this study, we successfully detected 11–17 perfect microsatellite loci in the *I. acutitarsus* species complex mitogenomes and 4–6 perfect microsatellite loci in the *I. ovatus* species complex mitogenomes, and found five microsatellite types, including mono-, di-, tri-, tetra-, and penta-nucleotides (Figure 5A). The relative abundances of mitochondrial microsatellite loci were 0.760–1.175 for the *I. acutitarsus* species complex, 0.276–0.414 for the *I. ovatus* species complex, respectively (Figure 5B). Recently, Chen et al. also found a large number of microsatellites in the *D. silvarum* mitogenome [96].

We also performed microsatellite sequence analysis for the Ixodidae family for the first time. A total of 917 and 4367 microsatellite loci, belonging to mono-, di-, tri-, tetra-, penta-, and hexa-nucleotides six microsatellite types, were detected from 83 *Ixodes* and 357 Ixodidae mitogenomes, respectively (Figure 5C). The counts of different microsatellite types varied greatly, with mononucleotides microsatellites accounting for the highest proportion, reaching 50.82% (466/917 for the *Ixodes* genus) and 44.08% (1925/4367 for the Ixodidae family), respectively, followed by trinucleotide microsatellites, reaching 16.03% (147/917 for the *Ixodes* genus) and 26.54% (1159/4367 for the Ixodidae family), respectively (Figure 5C). From the analysis of the microsatellite motif, there were 19 motifs in *Ixodes* mitogenomes and 28 motifs in Ixodidae mitogenomes; the main microsatellite motifs in both *Ixodes* and Ixodidae mitogenomes were mononucleotides A, followed by trinucleotides AAT (Figure 5C).

#### 3.2.3. Gene Rearrangement

Gene arrangement comparison is an important tool for resolving deep-level phylogenetic relationships [97,98]. Genome rearrangement analysis is a routine analysis in comparative mitochondrial genomics [33,72,73,99,100,101]. Regarding the Ixodidae mitogenomes, Black and Roehrdanz first reported two gene arrangement types [102], and Shao et al. further summed up three types [59], which were confirmed by another study [76]. Later, one unique gene arrangement model for the metastriate Ixodidae was described in *Amblyomma transversale* (now named *Africaniella transversale*) mitogenome [62]. Just recently, another novel arrangement *trnA*-*trnR*-*trnN*-*trnE*-*trnS1*-*trnF* was discovered in *I. fecialis* mitogenomes [103]. Additionally, Duan et al. reported that *Hy. asiaticum* had a novel genome arrangement *trnA*-*trnR*-*trnN*-*trnS1*-*ND1*-*trnE*-*trnL2*-*rrnL* that differs from the typical metastriate Ixodidae arrangement *trnA*-*trnR*-*trnN*-*trnS1*-*trnE*-*ND1*-*trnL2*-*rrnL* [71]; however, our analysis found that this novel arrangement type was caused by an annotation error of the *ND1* gene.

**Figure 6:**
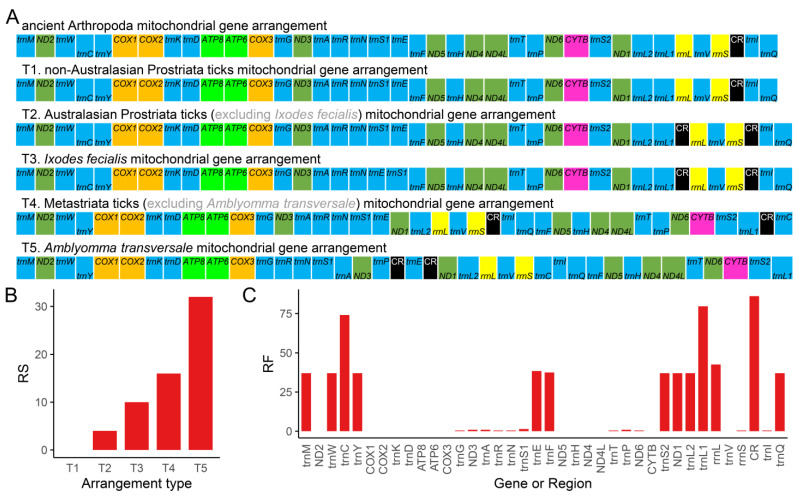
Mitochondrial gene arrangements of Ixodidae species. (**A**) The five mitochondrial gene arrangement types in Ixodidae group. (**B**) The rearrangement score (RS) of every gene arrangement type in Ixodidae group. (**C**) The rearrangement frequency (RF) of different mitochondrial genes or region in Ixodidae group. The tRNAs are abbreviated by the one-letter code for the corresponding amino acid, *trnL1* = *tRNA-Leu(CUN)*, *trnL2* = *tRNA-Leu(UUR)*, *trnS1* = *tRNA-Ser(AGN)*, and *trnS2* = *tRNA-Ser(UCN)*.

**Figure 7:**
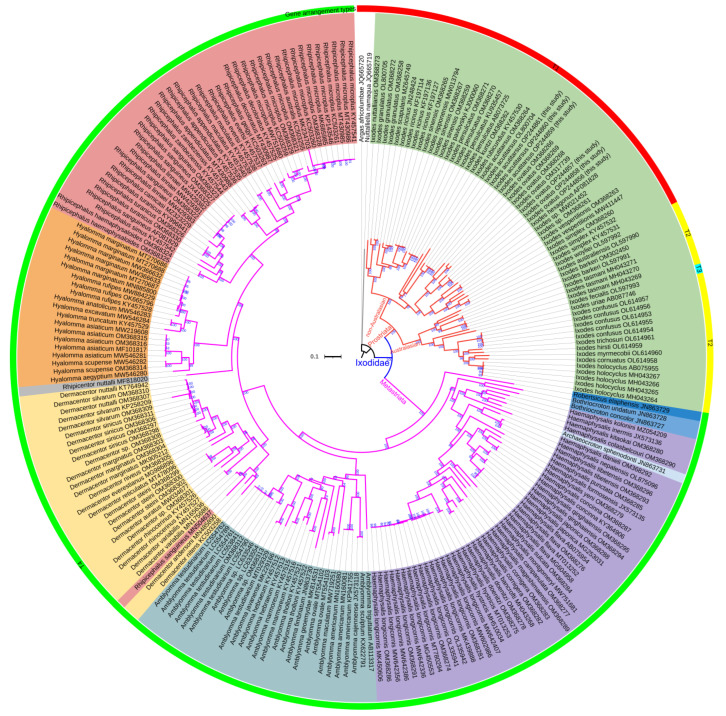
ML tree of Ixodidae species inferred from the concatenated DNA sequences of 15 mitochondrial genes. *A. africolumbae* and *N. namaqua* act as the outgroup. Ultrafast bootstrap percentage (UBP) is given at each node. The different colors of the outer ring represent different gene arrangement types in Figure 6.

In this study, we summarized five different gene arrangement types based on the comparison of all available Ixodidae complete mitogenomes to date (Figure 6A). The gene arrangement types T1, T2, and T3 were discovered in Prostriata ticks, while T4 and T5 were found in Metastriata ticks (Figure 7). Type T1 was identical to the ancient Arthropoda gene arrangement type found in *Limulus polyphemus* [58], which was assumed to be the ancestral type of arthropods and also found in Liphistiidae spiders [24,34], Nuttalliellidae ticks [104,105], and Argasidae ticks [59,104]. The non-Australasian Prostriata ticks had type T1, and almost all Australasian Prostriata ticks had type T2, except that *I. fecialis* owned type T3; Type T4 was the most common type, occurring in almost all Metastriata ticks, whereas type T5 only occurred in *Am. transversale* (now named *Af. transversale*). Among the five gene arrangement types, there were three types of gene position changes: duplication of CR, inversion of *trnC* and *trnP*, shuffling of many genes, and translocation of many genes. Compared to the ancient Arthropoda gene arrangement type, type T5 had the highest RS value (Figure 6B), indicating that it undergone the most complex changes. Furthermore, the RF value of 14 mitochondrial genes was only 0 (Figure 6C), indicating that their adjacent genes were not rearranged. The highest RF value was assigned to CR, followed by *trnL1* and *trnC*. We identified that the segment *trnY*-*COX1*-*trnS1*-*COX2*-*trnK*-*ATP8*-*ATP6*-*COX3*-*trnG* was the most conservative region, while the region near CR was the rearrangement hot spot in Ixodidae mitogenomes.

### 3.3. Mitogenomic Phylogenetic Analyses of Ixodidae Species

Unstable and uncertain phylogenetic relationships may affect our understanding of diversity and evolutionary history [25]. Molecular identification of existing and new species can be assisted by gene fragments (e.g., *rrnL* and *COX1*) [20,106], but complete mitogenomes may provide more accurate signals than gene fragments for phylogenetic reconstruction [23,33,61,73]. In this study, the phylogenetic analyses were conducted based on 13 PCGs and 2 rRNAs, which were concatenated into a sequence matrix of 12,892 bp representing 87% of the entire mitogenome alignments.

The ML and BI phylogenetic analyses yielded two highly similar topologies using the same sequence matrix (Figure 7 and Figure S3). Compared to previous studies [3,39], at the genus level, our study inferred more forceful phylogenetic relationships with stronger support (100% UBP, 1.00 BPP). Both ML and BI tree topologies delimited two lineages: Prostriata and Metastriata. The Prostriata lineage consisted of the single genus *Ixodes*; the Australasian *Ixodes* clade and the non-Australasian *Ixodes* clade were clustered with strong support (100% UBP, 1.00 BPP). The *Ixodes* genus was located at the basal position of the Ixodidae family, as in previous studies based on PCGs dataset [3,4,38,81,107], not clustered with the Argasidae ticks [36]. The tick *I. ovatus* parasitizing giant pandas clustered with the conspecific individuals infesting other animals, as *I. acutitarsus* did. The subclade ((*I. simplex* + *I. vespertilionis*) + *Ixodes* sp.) formed the sister-group relationship with *I. ovatus*, and *I. rubicundus* was the sister taxon of *I. acutitarsus*. The Metastriata lineage was further divided into eight clades with the relationship (*Robertsicus* + ((*Bothriocroton* + *Haemaphysalis*) *+* (*Amblyomma* + (*Dermacentor* + (*Rhipicentor* + (*Hyalomma* + *Rhipicephalus*)))))), as in previous mitochondrial PCGs phylogeny [3,7]. Most genera were fully supported as monophyletic groups, while *Haemaphysalis* genus was the paraphyletic group. *Ar. sphenodonti* was embedded in the *Haemaphysalis* clade with very strong support (100% UBP, 1.00 BPP), which was consistent with previous studies based on 13 mitochondrial PCGs [3,38]. It implied that the taxonomy of *Ar. sphenodonti* was debatable. At one time, *Ar. sphenodonti* was classified as *Amblyomma sphenodonti* [35]. Burger et al. found the polyphyly of the genus *Amblyomma* according to ML tree inferred from combined mitochondrial and nuclear DNA datasets, and thought that *Am. sphenodonti* and *Amblyomma elaphense* did not belong in the genus *Amblyomma* [108]. Later, they established two new monotypic genera, *Archaeocroton* and *Robertsicus*, for these two species [109]. Based on our phylogenetic inferences, we suggest that, in the future, an in-depth morphological comparison among *Ar. sphenodonti*, *Ha. colasbelcouri*, *Ha. kitaokai*, *Ha. kolonini*, *Ha. inermis*, and other *Haemaphysalis* species is necessary to determine whether *Ha. colasbelcouri*, *Ha. kitaokai*, *Ha. Kolonini*, and *Ha. inermis* could be assigned to the *Archaeocroton* genus or whether a new genus could be established for them.

## 4. Conclusions

In the present study, we obtained six complete mitogenomes of two *Ixodes* species parasitized on giant pandas, discussed the comparative mitogenomic characteristics with other available Ixodidae mitogenomes, and carried out the phylogenetic reconstruction of Ixodidae family. The newly determined mitogenomes of *I. ovatus* and *I. acutitarsus* were 14,539–14,543 bp and 14,472–14,473 bp, respectively. These six mitogenomes had the typical gene set comprising 22 tRNAs, 13 PCGs, two rRNAs, and one control region, and their gene arrangement type was the same as that of non-Australasian Prostriata ticks. In *I. ovatus* and *I. acutitarsus* mitogenomes, two Tick-Box motifs were found. We found that *I. ovatus* was a species complex with high genetic divergence, and thought that different groups of *I. ovatus* might represent distinct species. Comparative mitogenomic analyses revealed that the Ixodidae species had relatively high overall A + T content with a mean of 78.08%; their GC-skews of whole mitogenome were strongly negative, and the GC-skews of the light-chain-transcribed genes were higher than that of the heavy-chain-transcribed genes; their AT-skews of whole mitogenome fluctuated around 0, which deviated from the general characteristics of the metazoan mitogenomes. A large number of microsatellites such as (A)n and (AAT)n existed in Ixodidae mitogenomes. Five different mitochondrial gene arrangement types (T1–T5) were found, involving duplication of CR, inversion of *trnC*, and translocation of many genes. Type T4 was the most common type and was shared by almost all Metastriata ticks. The *trnY*-*COX1*-*trnS1*-*COX2*-*trnK*-*ATP8*-*ATP6*-*COX3*-*trnG* segment was the most conserved region, while the region near CR was the hotspot of mitochondrial genome rearrangements in Ixodidae. ML and BI phylogenetic analyses based on 13 PCGs and two rRNAs sequences showed great consistency in genus level relationships (*Ixodes* + (*Robertsicus* + ((*Bothriocroton* + *Haemaphysalis*) *+* (*Amblyomma* + (*Dermacentor* + (*Rhipicentor* + (*Hyalomma* + *Rhipicephalus*))))))) with strong support. Most genera were fully supported as monophyletic groups, while the *Haemaphysalis* genus was the paraphyletic group. *Ar. sphenodonti* was embedded in *Haemaphysalis* clade with very strong support. These results expand our knowledge of the diversity and evolution of Ixodidae mitogenomes, and provide more genetic information for control and prevention of tick diseases.

## Figures and Tables

**Table 1:** The pairwise K2P genetic distances for *I. ovatus* species complex based on the *rrnL* (upper right matrix) and *COX1* (lower left matrix) genes.

Group	Individual	*I. ovatus* OM368266	*I. ovatus* OM368268	*I. ovatus* OM368269	*I. ovatus* OM317739	*I. ovatus* OP244856	*I. ovatus* OP244857	*I. ovatus* OP244858
1	*I. ovatus* OM368266		0.1294	0.1403	0.1844	0.1982	0.1989	0.1972
2	*I. ovatus* OM368268	0.1549		0.0345	0.1945	0.1988	0.1974	0.1978
2	*I. ovatus* OM368269	0.1585	0.0457		0.1992	0.1936	0.1911	0.1925
3	*I. ovatus* OM317739	0.1894	0.1812	0.1729		0.0740	0.0740	0.0731
4	*I. ovatus* OP244856	0.1922	0.1608	0.1559	0.1098		0.0024	0.0008
4	*I. ovatus* OP244857	0.1896	0.1600	0.1534	0.1090	0.0046		0.0016
4	*I. ovatus* OP244858	0.1913	0.1600	0.1550	0.1091	0.0007	0.0039	

**Table 2:** The pairwise K2P genetic distances for *I. acutitarsus* species complex based on the *rrnL* (upper right matrix) and *COX1* (lower left matrix) genes.

Group	Individual	*I. acutitarsus* OM368264	*I. acutitarsus* OL800704	*I. acutitarsus* OP244859	*I. acutitarsus* OP244860	*I. acutitarsus* OP244861
1	*I. acutitarsus* OM368264		0.0502	0.0491	0.0491	0.0500
2	*I. acutitarsus* OL800704	0.0776		0.0024	0.0024	0.0016
2	*I. acutitarsus* OP244859	0.0775	0.0065		0	0.0024
2	*I. acutitarsus* OP244860	0.0775	0.0065	0		0.0024
2	*I. acutitarsus* OP244861	0.0754	0.0033	0.0046	0.0046	

## Data Availability

The complete mitogenome sequences obtained in this study are openly available in the GenBank database (https://www.ncbi.nlm.nih.gov/genbank/) under the accession IDs OP244856-OP244861.

## Supplementary Materials

The following supporting information can be downloaded at: https://www.mdpi.com/article/10.3390/genes13112049/s1, Table S1. The tick species and their corresponding mitogenomes used in this study; Table S2. The appropriate partition-specific substitution model used in both ML and BI analyses; Table S3. Annotations of the mitogenomes of *I. ovatus* and *I. acutitarsus*; Figure S1. Nucleotide composition of Ixodidae mitogenomes; Figure S2. AT-skew and GC-skew of Ixodidae mitochondrial PCGs and rRNAs; Figure S3. BI tree of Ixodidae species inferred from the concatenated DNA sequences of 15 mitochondrial genes. Bayesian posterior probability (BPP) is given at each node.
