# Supplementary material for: Complete Mitogenomes of Ticks Ixodes acutitarsus and Ixodes ovatus Parasitizing Giant Panda: Deep Insights into the Comparative Mitogenomic and Phylogenetic Relationship of Ixodidae Species
Source: Genes (Basel). 2022 Nov 6;13(11):2049. doi: 10.3390/genes13112049 (PMC9691169; doi:10.3390/genes13112049)
Supplement: Supplementary file 1 [file genes-13-02049-s001.zip › Supplementary File/Table S1~S3.pdf]

Table S1. The tick species and their corresponding mitochondrial genomes used in this study.

Genus	Species	GeneBank ID	Comparative mitogenomic analyses	Mitogenomic phylogenetic analyses	Mitogenome size (bp)	Gene arrangement type
**Ixodidae family**						
Ixodes	*Ixodes acutitarsus*	OL800704\NC_061225	√	√	14475	T1
		OM368264	√	√	14481	T1
		OP244859	√	√	14473	T1
		OP244860	√	√	14473	T1
		OP244861	√	√	14472	T1
	Ixodes australiensis	OL597990\NC_062625	√	√	15217	T2
	Ixodes barkeri	OL597991\NC_062626	√	√	15259	T2
		OM302450	√	√	15259	T2
	Ixodes confusus	OL614953\NC_062629	√	√	14939	T2
		OL614954	√	√	14941	T2
		OL614955	√	√	14944	T2
		OL614956	√	√	14939	T2
		OL614957	√	√	14942	T2
	Ixodes cornuatus	OL614958\NC_062630	√	√	14985	T2
	Ixodes fecialis	OL597992\NC_062628	√	√	15256	T3
	Ixodes granulatus	OL800705\NC_061226	√	√	14540	T1
		OM368258	√	√	14538	T1
		OM368272	√	√	14541	T1
	Ixodes hexagonus	AF081828\NC_002010	√	√	14539	T1
	Ixodes hirsti	OL614959\NC_062631	√	√	15040	T2
	Ixodes holocyclus	AB075955\NC_005293	√	√	15007	T2
		MH043264	√	√	15010	T2
		MH043265	√	√	15006	T2
		MH043266	√	√	15004	T2

		MH043267	√	√	15007	T2
	Ixodes kuntzi	OM368262\NC_062157	√	√	14524	T1
	Ixodes myrmecobii	OL614960\NC_062632	√	√	14995	T2
	Ixodes nipponensis	MW013794\NC_058242	√	√	14505	T1
	Ixodes nuttallianus	OM368273\NC_062062	√	√	14604	T1
	Ixodes ovatus	OM368266\NC_062061	√	√	14512	T1
		OM317739	√	√	14520	T1
		OM368268	√	√	14507	T1
		OM368269	√	√	14510	T1
		OP244856	√	√	14539	T1
		OP244857	√	√	14543	T1
		OP244858	√	√	14543	T1
	Ixodes pavlovskyi	KJ000060\NC_023831	√	√	14575	T1
	Ixodes persulcatus	AB073725\NC_004370	√	√	14539	T1
		KU935457	√	√	14539	T1
		OM368270	√	√	14550	T1
		OM368271	√	√	14545	T1
	Ixodes ricinus	JN248424\NC_018369	√	√	14566	T1
		KF197114	√	√	14575	T1
		KF197115	√		14579	T1
		KF197116	√		14572	T1
		KF197117	√		14567	T1
		KF197118	√		14578	T1
		KF197119	√		14578	T1
		KF197120	√		14578	T1
		KF197121	√		14571	T1
		KF197122	√		14570	T1
		KF197123	√		14579	T1
		KF197124	√		14573	T1
		KF197125	√		14575	T1

		KF197126	√		14574	T1
		KF197127	√	√	14578	T1
		KF197128	√		14578	T1
		KF197129	√		14570	T1
		KF197130	√		14575	T1
		KF197131	√		14573	T1
		KF197132	√		14574	T1
		KF197133	√		14578	T1
		KF197134	√		14571	T1
		KF197135	√		14574	T1
		KF197136	√	√	14578	T1
	Ixodes rubicundus	KY457530	√	√	14565	T1
	Ixodes scapularis	MZ645749	√	√	14537	T1
	Ixodes simplex	OM368260\NC_062060	√	√	14566	T1
		KY457531	√	√	14550	T1
		KY457532	√	√	14551	T1
	Ixodes sinensis	OM368259\NC_062059	√	√	14534	T1
		OM368265	√	√	14527	T1
		OM368267	√	√	14530	T1
	Ixodes sp.	MW021452	√	√	14543	T1
		OM368261	√	√	14545	T1
	Ixodes tasmani	MH043269\NC_041086	√	√	15227	T2
		MH043270	√	√	15163	T2
		MH043271	√	√	15219	T2
	Ixodes trichosuri	OL614961\NC_062633	√	√	15001	T2
	Ixodes uriae	AB087746\NC_006078	√	√	15053	T2
	Ixodes vespertilionis	MW411447\NC_058244	√	√	14559	T1
		OM368263	√	√	14548	T1
	Ixodes woyliei	OL597992\NC_062627	√	√	15062	T2
Robertsicus	*Robertsicus elaphensis*	JN863729\NC_017758	√	√	14627	T4

Bothriocroton	*Bothriocroton concolor*	JN863727\NC_017756	√	√	14809	T4
	Bothriocroton undatum	JN863728\NC_017757	√	√	14769	T4
Archaeocroton	*Archaeocroton sphenodonti*	JN863731\NC_017745	√	√	14772	T4
Haemaphysalis	*Haemaphysalis bancrofti*	MH043268\NC_041076	√	√	14673	T4
	Haemaphysalis campanulata	OM368277\NC_062159	√	√	14691	T4
	Haemaphysalis colasbelcouri	OM368290\NC_062164	√	√	14885	T4
	Haemaphysalis concinna	KY364906\NC_034785	√	√	14675	T4
		OM368287	√	√	14677	T4
	Haemaphysalis cornigera	OM368282\NC_062162	√	√	14681	T4
		OM368283	√	√	14680	T4
	Haemaphysalis danieli	OM368292\NC_062065	√	√	14739	T4
	Haemaphysalis doenitzi	OM368275\NC_062158	√	√	14671	T4
		OM368278	√	√	14673	T4
	Haemaphysalis flava	AB075954\NC_005292	√	√	14686	T4
		MG604958	√	√	14689	T4
		MT013252	√	√	14699	T4
		OM368276	√	√	14685	T4
	Haemaphysalis formosensis	JX573135\NC_020334	√	√	14676	T4
	Haemaphysalis hystricis	MH510034\NC_039765	√	√	14716	T4
		MT013253	√	√	14715	T4
	Haemaphysalis inermis	JX573136\NC_020335	√	√	14846	T4
	Haemaphysalis japonica	MG253031\NC_037246	√	√	14685	T4
		OM368288	√	√	14677	T4
	Haemaphysalis kitaokai	OM368280\NC_062161	√	√	14936	T4
	Haemaphysalis kolonini	MZ054209	√	√	14948	T4
	Haemaphysalis longicornis	MG450553\NC_037493	√	√	14718	T4
		MK450606	√	√	14694	T4
		MK439888	√	√	14693	T4
		MT780294	√	√	14706	T4
		OL335941	√	√	14694	T4

		OL335942	√	√	14694	T4
		OM368291	√	√	14694	T4
		OM368286	√	√	14694	T4
		OM368281	√	√	14693	T4
		OM368274	√	√	14693	T4
		MW602986	√	√	14694	T4
		MW642336	√	√	14694	T4
		MW642337	√		14694	T4
		MW642338	√		14694	T4
		MW642339	√		14694	T4
		MW642340	√		14694	T4
		MW642341	√		14694	T4
		MW642342	√		14694	T4
		MW642343	√		14694	T4
		MW642344	√		14694	T4
		MW642345	√		14694	T4
		MW642346	√		14694	T4
		MW642347	√		14694	T4
		MW642348	√		14694	T4
		MW642349	√		14694	T4
		MW642350	√		14694	T4
		MW642351	√		14694	T4
		MW642352	√		14694	T4
		MW642353	√		14694	T4
		MW642354	√		14694	T4
		MW642355	√		14694	T4
		MW642356	√	√	14694	T4
		MW642357	√		14694	T4
		MW642358	√		14694	T4
		MW642359	√		14694	T4

		MW642360	√		14694	T4
		MW642361	√		14694	T4
		MW642362	√		14694	T4
		MW642363	√		14694	T4
		MW642364	√		14694	T4
		MW642365	√		14694	T4
		MW642366	√		14694	T4
		MW642367	√		14694	T4
		MW642368	√		14694	T4
		MW642369	√		14691	T4
		MW642370	√		14693	T4
		MW642371	√		14693	T4
		MW642372	√		14693	T4
		MW642373	√		14693	T4
		MW642374	√		14693	T4
		MW642375	√		14693	T4
		MW642376	√		14693	T4
		MW642377	√		14693	T4
		MW642378	√		14693	T4
		MW642379	√		14693	T4
		MW642380	√		14693	T4
		MW642381	√		14693	T4
		MW642382	√		14693	T4
		MW642383	√		14693	T4
		MW642384	√		14693	T4
		MW642385	√		14693	T4
		MW642386	√	√	14693	T4
		MW642387	√		14693	T4
		MW642388	√		14693	T4
		MW642389	√		14693	T4

		MW642390	√		14693	T4
		MW642391	√		14696	T4
		MW642392	√		14696	T4
		MW642393	√		14696	T4
		MW642394	√		14696	T4
		MW642395	√		14695	T4
		MW642396	√		14695	T4
		MW642397	√		14695	T4
		MW642398	√		14695	T4
		MW642399	√		14695	T4
		MW642400	√		14696	T4
		MW642401	√		14695	T4
		MW642402	√		14692	T4
		MW642403	√		14692	T4
		MW642404	√		14692	T4
		MW642405	√		14692	T4
		MW642406	√		14692	T4
		MW642407	√	√	14692	T4
	Haemaphysalis mageshimaensis	OM368289\NC_062163	√	√	14721	T4
	Haemaphysalis montgomeryi	MW751681\NC_058312	√	√	14681	T4
	Haemaphysalis nepalensis	OL875096\NC_064124	√	√	14720	T4
	Haemaphysalis punctata	OM368285\NC_062064	√	√	14697	T4
	Haemaphysalis qinghaiensis	OM368294\NC_062067	√	√	14683	T4
		OM368295	√	√	14678	T4
	Haemaphysalis sulcata	OM368284\NC_062063	√	√	14679	T4
	Haemaphysalis tibetensis	OM368293\NC_062066	√	√	14715	T4
		OM368296	√	√	14714	T4
	Haemaphysalis yeni	OM368279\NC_062160	√	√	14690	T4
Amblyomma	*Amblyomma americanum*	KP941755\NC_027609	√	√	14709	T4
		MN160080	√	√	14708	T4

		MN160081	√	√	14708	T4
	Amblyomma cajennense	JX573118\NC_020333	√	√	14780	T4
	Amblyomma fimbriatum	JN863730\NC_017759	√	√	14705	T4
	Amblyomma geoemydae	MK814531	√	√	14780	T4
	Amblyomma hebraeum	KY457512	√	√	14657	T4
		KY457513	√	√	14654	T4
	Amblyomma javanense	MK229166\NC_043872	√	√	14780	T4
	Amblyomma maculatum	MW719251	√	√	14803	T4
	Amblyomma marmoreum	KY457515	√	√	14676	T4
		KY457516	√	√	14677	T4
	Amblyomma ovale	MT554102\NC_050255	√	√	14760	T4
		MT554103	√	√	14576	T4
	Amblyomma sculptum	KX622791\NC_032369	√	√	14780	T4
	Amblyomma sp.	OM368313	√	√	14760	T4
		LC633546	√	√	14832	T4
		LC633547	√		14831	T4
		LC633548	√		14836	T4
		LC633549	√		14838	T4
		LC633550	√		14835	T4
		LC633551	√		14834	T4
		LC633552	√		14830	T4
		LC633553	√		14834	T4
		LC633554	√	√	14836	T4
	Amblyomma testudinarium	OM368312\NC_062071	√	√	14976	T4
		LC553841	√	√	14835	T4
		MT029329	√	√	14760	T4
		LC554761	√	√	14835	T4
		LC554762	√	√	14835	T4
		LC554763	√		14837	T4
		LC554764	√		14839	T4

		LC554765	√		14835	T4
		LC554766	√		14834	T4
		LC554767	√		14836	T4
		LC554768	√		14835	T4
		LC554769	√		14834	T4
		LC554770	√		14835	T4
		LC554771	√		14835	T4
		LC554772	√		14835	T4
		LC554773	√		14837	T4
		LC554774	√		14837	T4
		LC554775	√		14834	T4
		LC554776	√		14836	T4
		LC554777	√		14835	T4
		LC554778	√		14835	T4
		LC554779	√		14833	T4
		LC554780	√		14835	T4
		LC554781	√		14835	T4
		LC554782	√		14831	T4
		LC554783	√		14835	T4
		LC554784	√		14832	T4
		LC554785	√		14830	T4
		LC554786	√		14830	T4
		LC554787	√		14835	T4
		LC554788	√		14836	T4
		LC554789	√	√	14835	T4
		LC554790	√	√	14834	T4
	Amblyomma tholloni	KY457521	√	√	14642	T4
		KY457522	√	√	14640	T4
	Amblyomma transversale	MT371795			15288	T5
		MT371796			15294	T5

	Amblyomma triguttatum	AB113317\NC_005963	√	√	14740	T4
Dermacentor	*Dermacentor andersoni*	MN485890\NC_061057	√	√	14806	T4
	Dermacentor auratus	MW034677\NC_059724	√	√	14766	T4
	Dermacentor everestianus	MG986896\NC_042764	√	√	15191	T4
	Dermacentor marginatus	OM368303\NC_062069	√	√	15178	T4
		MK905212	√	√	15067	T4
		OM368304	√	√	15177	T4
	Dermacentor nitens	KC503258\NC_023349	√	√	14839	T4
	Dermacentor niveus	OM368305\NC_062070	√	√	15110	T4
	Dermacentor nuttalli	KT764942\NC_028528	√	√	15086	T4
		OM368307	√	√	15086	T4
	Dermacentor reticulatus	MT478096	√	√	14806	T4
	Dermacentor rhinocerinus	KY457527	√	√	14708	T4
		KY457526	√	√	14708	T4
	Dermacentor silvarum	KP258209\NC_026552	√	√	14945	T4
		OM368310	√	√	15171	T4
		OM368309	√	√	15086	T4
	Dermacentor sinicus	OM368297\NC_062165	√	√	14996	T4
		OM368298	√	√	14997	T4
		OM368311	√	√	14947	T4
		OM368306	√	√	14991	T4
	Dermacentor sp.	OM368301	√	√	14796	T4
		OM368308	√	√	15307	T4
	Dermacentor steini	OM368299\NC_062068	√	√	14773	T4
		OM368300	√	√	14785	T4
		OM368302	√	√	14767	T4
	Dermacentor variabilis	MN165636\NC_061217	√	√	14837	T4
		MN175686	√	√	14832	T4
Rhipicentor	*Rhipicentor nuttalli*	MF818020\NC_039828	√	√	14779	T4
Hyalomma	*Hyalomma aegyptium*	MW546280	√	√	14707	T4

	Hyalomma anatolicum	MW546283	√	√	14731	T4
	Hyalomma asiaticum	MF101817	√	√	14720	T4
		OM368315	√	√	14723	T4
		OM368316	√	√	14723	T4
		MW546281	√	√	14722	T4
		MW219608\NC_053941	√	√	14723	T4
	Hyalomma excavatum	MW546284	√	√	14733	T4
	Hyalomma marginatum	MT270686\NC_056189	√	√	14764	T4
		MT270687	√	√	14762	T4
		MT270688	√	√	14763	T4
		MN885800	√	√	14764	T4
		MW366628	√		14762	T4
		MW366629	√		14762	T4
		MW366630	√		14762	T4
		MW366631	√		14761	T4
		MW366632	√	√	14765	T4
		MW366633	√	√	14765	T4
	Hyalomma rufipes	MW884229	√	√	14761	T4
		KY457528	√	√	14748	T4
		OK665796	√	√	14787	T4
	Hyalomma scupense	OM368314\NC_062166	√	√	14721	T4
		MW546282	√	√	14721	T4
	Hyalomma truncatum	KY457529	√	√	14731	T4
Rhipicephalus	*Rhipicephalus appendiculatus*	MT430988\NC_052829	√	√	15001	T4
		KY457535	√	√	14733	T4
		KY457536	√	√	14733	T4
	Rhipicephalus australis	KC503255\NC_023348	√	√	14891	T4
	Rhipicephalus camicasi	MZ323229\NC_061616	√	√	14725	T4
	Rhipicephalus decoloratus	MT430987\NC_052828	√	√	15268	T4
		KY457525	√	√	14782	T4

	Rhipicephalus evertsi	KY457537	√	√	14739	T4
		KY457538	√	√	14740	T4
	Rhipicephalus geigyi	KC503263\NC_023350	√	√	14948	T4
	Rhipicephalus haemaphysaloides	OM368324\NC_062072	√	√	14744	T4
		OM368325	√	√	14745	T4
	Rhipicephalus linnaei	MW429381\NC_060409	√	√	14711	T4
		MW429382	√	√	14717	T4
		MW429383	√	√	14715	T4
	Rhipicephalus maculatus	KY457539	√	√	14714	T4
		KY457540	√	√	14714	T4
	Rhipicephalus microplus	KC503261\NC_023335	√	√	14905	T4
		KC503260	√	√	14903	T4
		KC503259	√	√	14864	T4
		KP143546	√	√	15167	T4
		MK685985	√	√	15163	T4
		MK234703	√	√	14903	T4
		OM368321	√	√	14900	T4
		OM368328	√	√	14899	T4
		OM368329	√	√	14863	T4
		MW751680	√	√	14763	T4
		KY457541	√	√	15169	T4
		MT430986	√	√	14910	T4
		MT430985	√	√	14909	T4
	Rhipicephalus sanguineus	AF081829\NC_002074	√	√	14710	T4
		JX416325	√	√	14714	T4
		MN504637	√	√	14741	T4
		OM368322	√	√	14713	T4
		OM368323	√	√	14714	T4
		OM368327	√	√	14711	T4
	Rhipicephalus simus	KY457542	√	√	14721	T4

	Rhipicephalus turanicus	KY996841\NC_035946	√	√	14717	T4
		OM368326	√	√	14719	T4
		OM368330	√	√	14717	T4
	Rhipicephalus zambeziensis	KY457543	√	√	14691	T4
		KY457544	√	√	14691	T4
**Nuttalliellidae family**						
Nuttalliella	*Nuttalliella namaqua*	JQ665719		√	14425	T1
**Argasidae family**						
Argas	*Argas africolumbae*	JQ665720		√	14440	T1

Table S2. The appropriate partition-specific substitution model used in both ML and BI analyses.

Phylogenetic analysis	Charset	Partition	Model
**ML**	COX1_COX2	1-1533 2530-3201	GTR+F+I+G4
	ND1_ND2_ND4_ND5_ND4L	1534-2529 3202-4197 5296-6585 6586-8232 10495-10764	GTR+F+R6
	COX3	4198-4971	GTR+F+I+G4
	ND3_ATP6	4972-5295 8233-8886	GTR+F+R5
	ND6_ATP8	8887-9333 9334-9432	TVM+F+R6
	CYTB	9433-10494	GTR+F+I+G4
	rrnL_rrnS	10765-12125 12126-12892	TVM+F+R5
**BI**	COX1_COX2	1-1533 2530-3201	GTR+F+I+G4
	ND1_ND2_ND4	1534-2529 3202-4197 5296-6585	GTR+F+I+G4
	COX3	4198-4971	GTR+F+I+G4
	ND3_ATP6	4972-5295 8233-8886	GTR+F+I+G4
	ND5_ND4L	6586-8232 10495-10764	GTR+F+I+G4
	ND6_ATP8	8887-9333 9334-9432	GTR+F+I+G4
	CYTB	9433-10494	GTR+F+I+G4
	rrnL_rrnS	10765-12125 12126-12892	GTR+F+I+G4

Table S3. Annotations of the mitogenomes of *I. ovatus* and *I. acutitarsus* .

Gene	Product	***Ixodes ovatus* OP244856**			***Ixodes ovatus* OP244857**			***Ixodes ovatus* OP244858**			***Ixodes acutitarsus* OP244859**			***Ixodes acutitarsus* OP244860**			***Ixodes acutitarsus* OP244861**		
		**Location**	**Size**	**Start/Stop Codon**	**Location**	**Size**	**Start/Stop Codon**	**Location**	**Size**	**Start/Stop Codon**	**Location**	**Size**	**Start/Stop Codon**	**Location**	**Size**	**Start/Stop Codon**	**Location**	**Size**	**Start/Stop Codon**
trnM(cau)	tRNA-Met	1-67:+	67		1-68:+	68		1-68:+	68		1-65:+	65		1-65:+	65		1-65:+	65	
ND2	NADH dehydrogenase subunit 2	68-1037:+	970	ATT/T	69-1038:+	970	ATA/T	69-1038:+	970	ATT/T	66-1035:+	970	ATA/T	66-1035:+	970	ATA/T	66-1035:+	970	ATA/T
trnW(uca)	tRNA-Trp	1038-1102:+	65		1039-1103:+	65		1039-1103:+	65		1036-1099:+	64		1036-1099:+	64		1036-1099:+	64	
trnC(gca)	tRNA-Cys	1095-1156:-	62		1096-1157:-	62		1096-1157:-	62		1092-1155:-	64		1092-1155:-	64		1092-1155:-	64	
trnY(gua)	tRNA-Tyr	1161-1226:-	66		1162-1227:-	66		1162-1227:-	66		1157-1221:-	65		1157-1221:-	65		1157-1221:-	65	
COX1	cytochrome c oxidase subunit I	1219-2757:+	1539	ATT/TAA	1220-2758:+	1539	ATT/TAA	1220-2758:+	1539	ATT/TAA	1214-2752:+	1539	ATT/TAA	1214-2752:+	1539	ATT/TAA	1214-2752:+	1539	ATT/TAA
COX2	cytochrome c oxidase subunit II	2761-3436:+	676	ATG/T	2762-3437:+	676	ATG/T	2762-3437:+	676	ATG/T	2756-3431:+	676	ATG/T	2756-3431:+	676	ATG/T	2756-3431:+	676	ATG/T
trnK(cuu)	tRNA-Lys	3437-3507:+	71		3438-3508:+	71		3438-3508:+	71		3432-3498:+	67		3432-3498:+	67		3432-3498:+	67	
trnD(guc)	tRNA-Asp	3507-3572:+	66		3508-3573:+	66		3508-3573:+	66		3498-3559:+	62		3498-3559:+	62		3498-3559:+	62	
ATP8	ATP synthase F0 subunit 8	3573-3728:+	156	ATT/TAA	3574-3729:+	156	ATT/TAA	3574-3729:+	156	ATT/TAA	3560-3715:+	156	ATC/TAA	3560-3715:+	156	ATC/TAA	3560-3715:+	156	ATC/TAA
ATP6	ATP synthase F0 subunit 6	3722-4387:+	666	ATG/TAA	3723-4388:+	666	ATG/TAA	3723-4388:+	666	ATG/TAA	3709-4374:+	666	ATG/TAA	3709-4374:+	666	ATG/TAA	3709-4374:+	666	ATG/TAA
COX3	cytochrome c oxidase subunit III	4394-5171:+	778	ATG/T	4395-5172:+	778	ATG/T	4395-5172:+	778	ATG/T	4379-5156:+	778	ATG/T	4379-5156:+	778	ATG/T	4379-5156:+	778	ATG/T
trnG(ucc)	tRNA-Gly	5172-5233:+	62		5173-5234:+	62		5173-5235:+	63		5157-5222:+	66		5157-5222:+	66		5157-5222:+	66	
ND3	NADH dehydrogenase subunit 3	5234-5569:+	336	ATA/TAA	5235-5570:+	336	ATA/TAA	5236-5571:+	336	ATA/TAA	5223-5558:+	336	ATG/TAA	5223-5558:+	336	ATG/TAA	5223-5558:+	336	ATG/TAA
trnA(ugc)	tRNA-Ala	5573-5635:+	63		5574-5636:+	63		5575-5637:+	63		5564-5624:+	61		5564-5624:+	61		5564-5624:+	61	
trnR(ucg)	tRNA-Arg	5635-5697:+	63		5636-5698:+	63		5638-5700:+	63		5624-5687:+	64		5624-5687:+	64		5624-5687:+	64	
trnN(guu)	tRNA-Asn	5698-5764:+	67		5699-5765:+	67		5703-5769:+	67		5687-5752:+	66		5687-5752:+	66		5687-5752:+	66	
trnS1(ucu)	tRNA-Ser	5765-5819:+	55		5766-5820:+	55		5770-5824:+	55		5754-5809:+	56		5754-5809:+	56		5753-5808:+	56	
trnE(uuc)	tRNA-Glu	5832-5892:+	61		5834-5894:+	61		5839-5899:+	61		5810-5870:+	61		5810-5870:+	61		5809-5869:+	61	
trnF(gaa)	tRNA-Phe	5891-5956:-	66		5893-5958:-	66		5898-5963:-	66		5869-5930:-	62		5869-5930:-	62		5868-5929:-	62	
ND5	NADH dehydrogenase subunit 5	5957-7625:-	1669	ATT/T	5959-7627:-	1669	ATT/T	5964-7632:-	1669	ATT/T	5931-7596:-	1666	ATT/T	5931-7596:-	1666	ATT/T	5930-7595:-	1666	ATT/T
trnH(gug)	tRNA-His	7626-7689:-	64		7628-7691:-	64		7633-7696:-	64		7597-7658:-	62		7597-7658:-	62		7596-7657:-	62	
ND4	NADH dehydrogenase subunit 4	7692-9014:-	1323	ATG/TAA	7694-9016:-	1323	ATG/TAA	7699-9021:-	1323	ATG/TAA	7667-8983:-	1317	ATG/TAA	7667-8983:-	1317	ATG/TAA	7666-8982:-	1317	ATG/TAA
ND4L	NADH dehydrogenase subunit 4L	9008-9283:-	276	ATG/TAG	9010-9285:-	276	ATG/TAG	9015-9290:-	276	ATG/TAG	8977-9252:-	276	ATG/TAG	8977-9252:-	276	ATG/TAG	8976-9251:-	276	ATG/TAG
trnT(ugu)	tRNA-Thr	9286-9348:+	63		9288-9350:+	63		9293-9355:+	63		9255-9316:+	62		9255-9316:+	62		9254-9315:+	62	
trnP(ugg)	tRNA-Pro	9349-9415:-	67		9351-9417:-	67		9356-9422:-	67		9317-9379:-	63		9317-9379:-	63		9316-9378:-	63	
ND6	NADH dehydrogenase subunit 6	9403-9849:+	447	ATA/TAA	9405-9851:+	447	ATA/TAA	9410-9856:+	447	ATA/TAA	9382-9813:+	432	ATA/TAA	9382-9813:+	432	ATA/TAA	9381-9812:+	432	ATA/TAA
CYTB	cytochrome b	9849-10929:+	1081	ATG/T	9851-10931:+	1081	ATG/T	9856-10936:+	1081	ATG/T	9813-10893:+	1081	ATG/T	9813-10893:+	1081	ATG/T	9812-10892:+	1081	ATG/T
trnS2(uga)	tRNA-Ser	10930-10992:+	63		10932-10994:+	63		10937-10999:+	63		10894-10956:+	63		10894-10956:+	63		10893-10955:+	63	
ND1	NADH dehydrogenase subunit 1	11002-11929:-	928	ATG/T	11004-11931:-	928	ATG/T	11009-11936:-	928	ATG/T	10965-11892:-	928	ATG/T	10965-11892:-	928	ATG/T	10964-11891:-	928	ATG/T
trnL2(uaa)	tRNA-Leu	11931-11996:-	66		11933-11998:-	66		11938-12003:-	66		11893-11957:-	65		11893-11957:-	65		11892-11956:-	65	
trnL1(uag)	tRNA-Leu	12014-12073:-	60		12016-12075:-	60		12019-12078:-	60		11958-12017:-	60		11958-12017:-	60		11957-12016:-	60	
rrnL	16S ribosomal RNA	12055-13310:-	1256		12057-13314:-	1258		12060-13316:-	1257		11999-13240:-	1242		11999-13240:-	1242		11998-13239:-	1242	
trnV(uac)	tRNA-Val	13305-13366:-	62		13309-13370:-	62		13311-13372:-	62		13222-13283:-	62		13222-13283:-	62		13221-13282:-	62	
rrnS	12S ribosomal RNA	13367-14070:-	704		13371-14075:-	705		13373-14075:-	703		13284-13994:-	711		13284-13994:-	711		13283-13993:-	711	
A+T rich region	control region	14071-14404:+	334		14076-14408:+	333		14076-14408:+	333		13995-14337:+	343		13995-14337:+	343		13994-14336:+	343	
trnI(gau)	tRNA-Ile	14405-14469:+	65		14409-14473:+	65		14409-14473:+	65		14338-14401:+	64		14338-14401:+	64		14337-14400:+	64	
trnQ(uug)	tRNA-Gln	14467-14535:-	69		14471-14539:-	69		14471-14539:-	69		14399-14466:-	68		14399-14466:-	68		14398-14465:-	68	
