# Supplementary figures and images for: Complete Mitogenomes of Ticks Ixodes acutitarsus and Ixodes ovatus Parasitizing Giant Panda: Deep Insights into the Comparative Mitogenomic and Phylogenetic Relationship of Ixodidae Species
Source: Genes (Basel). 2022 Nov 6;13(11):2049. doi: 10.3390/genes13112049 (PMC9691169; doi:10.3390/genes13112049)

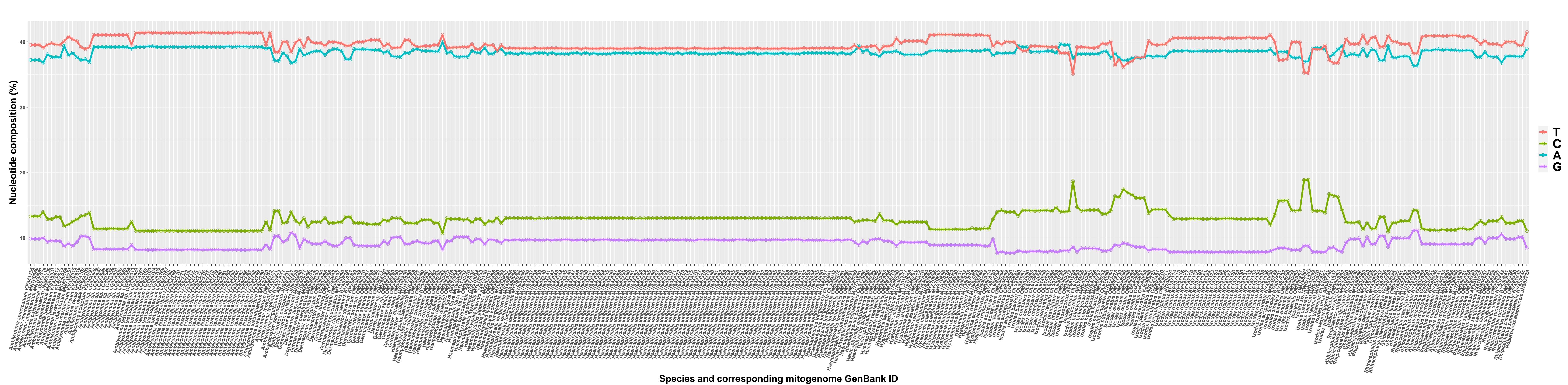

## Species and corresponding mitogenome GenBank ID

Supplement: Supplementary file 1 [file genes-13-02049-s001.zip › Supplementary File/Figure S1. Nucleotide composition of Ixodidae mitogenomes.pdf]

AT-skew

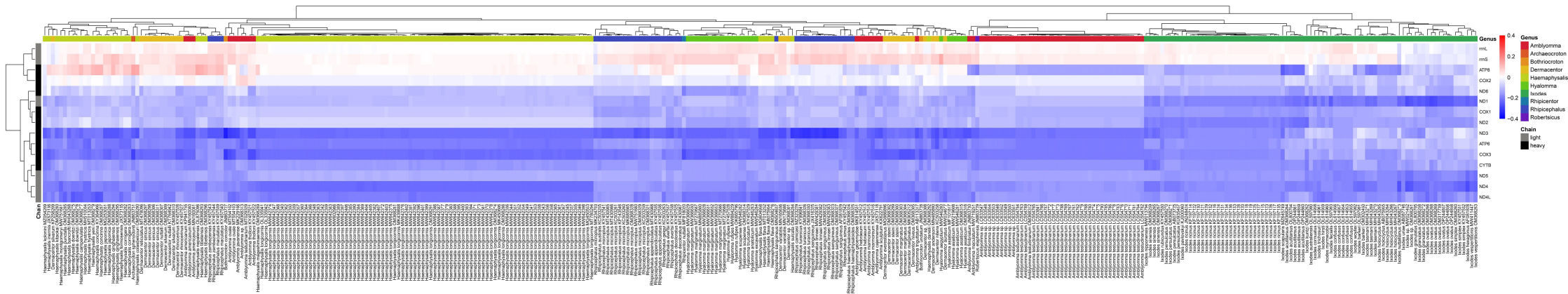

GC-skew

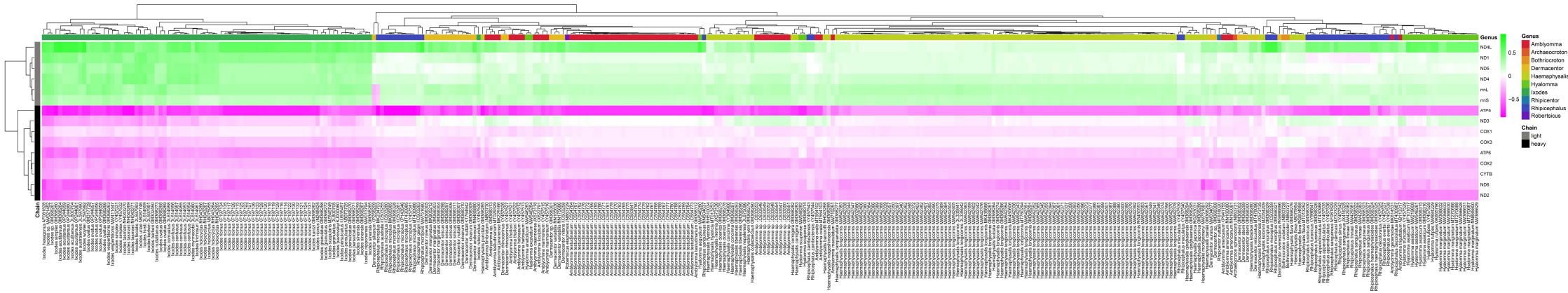

Supplement: Supplementary file 1 [file genes-13-02049-s001.zip › Supplementary File/Figure S2. AT-skew and GC-skew of Ixodidae mitochondrial PCGs and rRNAs.pdf]

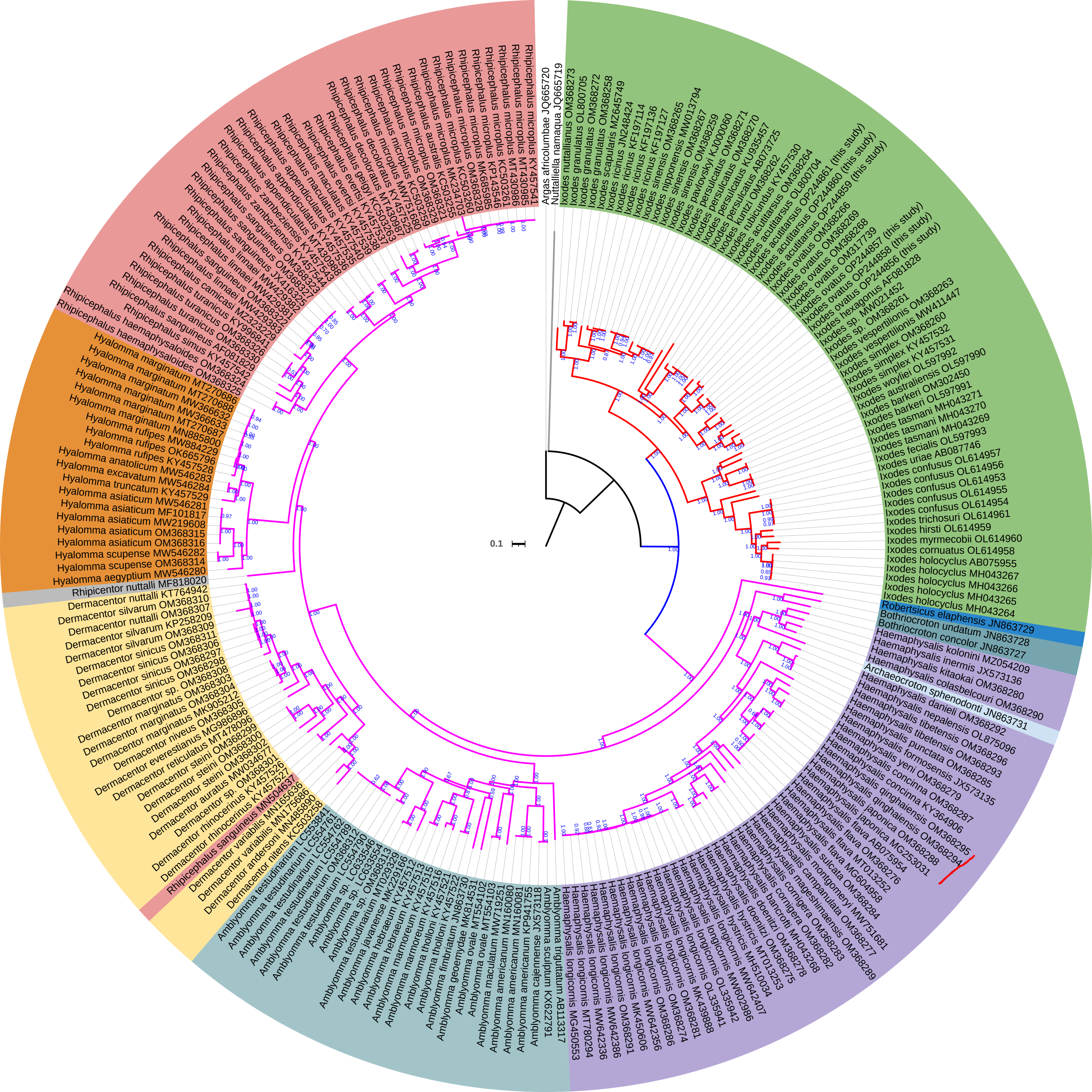

Supplement: Supplementary file 1 [file genes-13-02049-s001.zip › Supplementary File/Figure S3. BI tree of Ixodidae species inferred from the concatenated DNA sequences of 15 mitochondrial genes.pdf]
